# Sustained Spatial Attention in Touch: Modality-Specific and Multimodal Mechanisms

**DOI:** 10.1100/tsw.2011.34

**Published:** 2011-01-18

**Authors:** Chiara F. Sambo, Bettina Forster

**Affiliations:** ^1^ Institute of Psychiatry, King's College London, UK; ^2^ Psychology Department, City University London, UK

**Keywords:** spatial attention, touch, multimodal, body posture, event-related potentials (ERPs), functional magnetic resonance imaging (fMRI), positron emission tomography (PET)

## Abstract

Sustained attention to a body location results in enhanced processing of tactile stimuli presented at that location compared to another unattended location. In this paper, we review studies investigating the neural correlates of sustained spatial attention in touch. These studies consistently show that activity within modality-specific somatosensory areas (SI and SII) is modulated by sustained tactile-spatial attention. Recent evidence suggests that these somatosensory areas may be recruited as part of a larger cortical network,also including higher-level multimodal regions involved in spatial selection across modalities. We discuss, in turn, the following multimodal effects in sustained tactile-spatial attention tasks. First, cross-modal attentional links between touch and vision, reflected in enhanced processing of task-irrelevant visual stimuli at tactuallyattended locations, are mediated by common (multimodal) representations of external space. Second, vision of the body modulates activity underlying sustained tactile-spatial attention, facilitating attentional modulation of tactile processing in between-hand (when hands are sufficiently far apart) and impairing attentional modulation in within-hand selection tasks. Finally, body posture influences mechanisms of sustained tactile-spatial attention, relying, at least partly, on remapping of tactile stimuli in external, visuallydefined, spatial coordinates. Taken together, the findings reviewed in this paper indicate that sustained spatial attention in touch is subserved by both modality-specific and multimodal mechanisms. The interplay between these mechanisms allows flexible and efficient spatial selection within and across sensory modalities.

## INTRODUCTION

Unexpected, salient stimuli in the environment and on our skin (e.g., a bee on the back of our hand) can capture our attention without our intention. However, we are also able to voluntarily direct attention to a specific event or spatial location in order to select the most relevant information for current goals. This endogenous control of attention is crucial for everyday tasks. Indeed, researchers have long established that our sensory systems (e.g., visual, somatosensory) have limited capacity[1,2,3]. As a result, at any given time, we become aware of only a subset of the incoming sensory inputs. Selective attention allows us to process relevant information more thoroughlythan irrelevant stimuli in all sensory modalities. The neural underpinnings of this effect have been addressed by using an array of attentional paradigms and methodologies[4,5,6]. Although most of the research on the effects of attention on sensory processing has focused on the visual and auditory modalities[7,8,9], the last 2 decades have seen an increase of interest in the brain mechanisms underlying the attentional modulation of somatosensory responses (see [10,11]for reviews of behavioral studies on tactile attention). A number of these studies have focused on the neural correlates of tactile-*spatial*attention[3,11]. This field of investigation also has clinical implications because attentional biases to the body have been suggested to play an important role for the maintenance of medically unexplained (bodily) symptoms and chronic pain states[12,13,14].

Endogenous tactile-spatial attention has been experimentally manipulated either by cueing the to-be-attended body location on a trial-by-trial basis (*transient* attention) or by instructing participants to maintain their attention on one body part (e.g., the hand) for a longer time, usually for an entire experimental block (*sustained* attention). This paper reviews research to date on the neural mechanisms involved in sustained tactile-spatial attention. Several everyday situations require sustained attention to a certain body site; for example, when we explore the environment with our hands searching for something, we may focus attention on our hands for several minutes even without looking at them (covert attention). Despite this observation, there has been limited research on the neural substrates of sustained spatial attention in touch. Furthermore, most of these studies used event-related potentials (ERPs) to investigate the time course of the attentional modulation of tactile processing, while few of these studies have employed hemodynamic techniques (e.g., functional magnetic resonance imaging, fMRI, and positron emission tomography, PET), which provide higher spatial resolution.

Early investigations showed that sustained tactile-spatial attention modulates activity within somatosensory areas (SI and SII), facilitating processing of tactile stimuli[15,16,17]. While these studies investigated the tactile modality alone, in the last decade, researchers have been increasingly interested in how such attentional effects are modulated by other factors, such as multimodal input and body posture[18,19,20]. In particular, research has shown reciprocal influences between touch and vision during sustained tactile-spatial attention. In this paper, we review evidence on modality-specific and multimodal or supramodal effects, including unpublished data from our studies, and we discuss possible mechanisms underlying these effects. Throughout the paper, we use the terms: (a) “modality-specific” to refer to effects associated with activation of brain areas specifically dedicated to processing information in one sensory modality (e.g., somatosensory cortex); (b) “multimodal” to indicate effects that involve more than one sensory modality at a time (e.g., touch and vision) and possibly result from activation of bimodal, or trimodal, neurons in the so-called “multimodal” areas (e.g.,intraparietal sulcus, IPS[21]); and (c) “supramodal” to indicate effects that are mediated by a cortical network, including frontoparietal areas, whichare common across sensory modalities and may be activated by one modality at a time.

## NEURAL MECHANISMS UNDERLYING SUSTAINED TACTILE-SPATIAL ATTENTION

In the last few decades, ERPs have been increasingly used to gain insights into the time course of attentional processing. ERPs consist of successive positive- and negative-going deflections in the EEG activity, timelocked to an event (e.g., stimulus presentation). Early investigations on sustained tactile-spatial attention have compared ERPs elicited by electrical stimuli applied to the hands, while the participants’ attention was either directed to or away from the stimulated hand for an entire block of stimuli[15,16,17,22]. In all these studies, the participants’ task was to silently count stimuli delivered at the currently attended hand. These studies have consistently shown that attending to the location of a tactile stimulus modulates early- and mid-latency somatosensory components of ERPs (i.e., N80[17],

P100[15], and N140[15,16,17,22]), as reflected in enhanced amplitudes for stimuli presented at attended compared to unattended locations. These effects are followed by a late positive component (LPC), elicited from about 200 msecpoststimulus onset, which is enhanced for tactile stimuli at attended locations[15,16,17]. Taken together, these studies show that selection of tactile information occurs from the early stages of processing. In particular, short- and mid-latency ERP components are thought to reflect modality-specific processing, thus indicating that sustained spatial attention can affect perceptualstages of tactile processing[23]. In addition, the LPC may indicate attentional influences at postperceptual processing levels[16,24]. Thus, spatial attention appears to affect neural activities related to both processing of physical attributes of stimuli and stimulus identification and categorization.

The findings discussed so far have been partly confirmed by more recent ERP studies that used mechanical tactile stimuli and required participants to make a vocal or motor response to tactile targets[25,26,27]. These studies reported that sustained attention to one hand affected early- and mid-latency somatosensory components (i.e., N80[26], P100[26,27], and N140[25,26,27]), as well as later stages of processing (see Fig. 1[25]). However, unlike earlier investigations, this late attentional modulation was more negative for tactile stimuli at attended compared to unattended locations (“negative difference”, Nd). This reversed effect at later stages of processing may reflect differences in experimental procedures, including working memory load, which is higher in tasks requiring mental count of target stimuli throughout a block as compared to vocal or foot response to all targets (see [28] for a discussion). Taken together, these findings tally with previous evidence that sustained tactile-spatial attention influences both perceptual processing and decision-making stages.

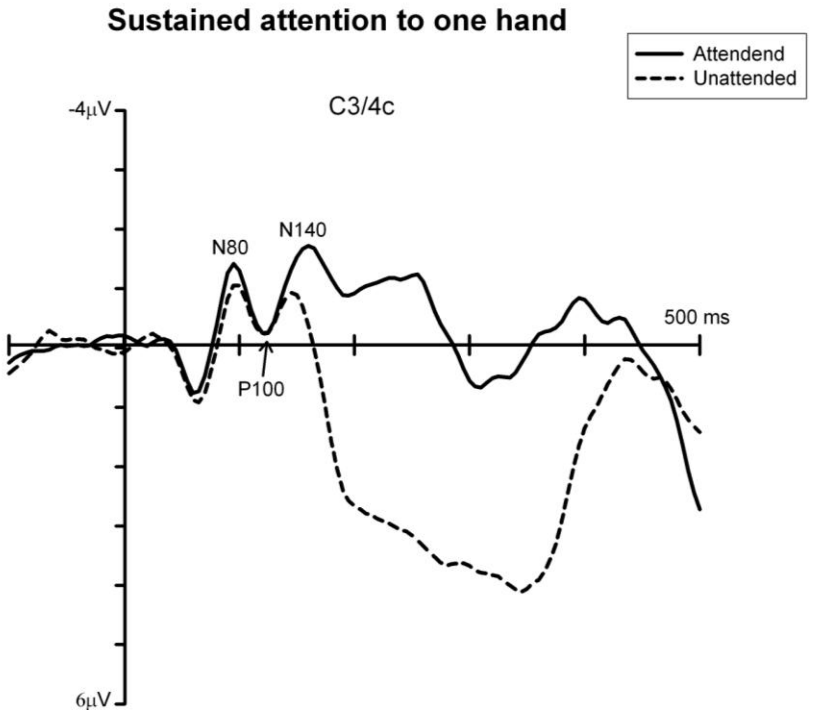

**FIGURE 1.**Effects of sustained spatial attention on tactile processing.The figure shows grand-averaged somatosensory ERPs elicited in the 500-msec interval following stimulus onset by tactile (mechanical) stimuli at attended (*solid lines*) and unattended (*dashed lines)* locations. Somatosensory ERPs are shown for electrode C3/4 (i.e., over somatosensory cortex) contralateral to the site of tactile stimulation. ERPs were significantly enhanced for tactile stimuli presented at attended vs.

unattended locations in the range of the N140 component and at later latencies (Nd). (Data from Forster and Eimer[25].)

Furthermore, these studies provide additional insights into the mechanisms involved in sustained tactile-spatial attention by comparing different types of tactile selection. For example, Eimer and Forster[26] and Zopf and colleagues[27] found evidence for a partial dissociation between neural mechanisms underlying sustained and transient allocation of spatial attention in touch[25,27]. These differences were found at early stages of somatosensory processing in both studies. Specifically, Eimer and Forster[26] found that the N80 and P100 components were uniquely modulated by sustained and transient attention, respectively; and in Zopt et al.'s study[27], the topography of the P100 component was found to differ between sustained and transient attention conditions, with attentional modulations being observed ipsilaterally and contralaterally to the site of tactile stimulation, respectively. In addition, both these studies found that subsequent stages of tactile processing (i.e., overlapping with the N140 component and Nd) were similarly modulated by sustained and transient attention. Together, these results suggest that sustained and transient attentional orienting in space may differ at early (perceptual), but not later, stages of tactile processing.

On the other hand, sustained attention to a body location has been shown to modulate somatosensory ERPs at similar latencies as does "nonspatial" sustained attention (i.e., selection of nonspatial attributes of tactile stimuli, e.g., intensity)[25], thus suggesting that selection of spatial and nonspatial tactile information may occur in parallel (see also [29,30,31,32]for evidence in support of similar "where" and "what" dissociations in somatosensory processing in nonattentional tasks). Interestingly, while spatial and nonspatialattentional selection has been also shown to operate in parallel in the auditory modality, similar to touch[33], in vision, selection of nonspatial attributes of stimuli is hierarchically dependent on selection of spatial information[34].

The studies reviewed so far show that early- and mid-latency somatosensory components (i.e., N80, P100, and N140) can be modulated by the sustained focus of spatial attention, with some differences in the specific components involved, possibly reflecting the different methods and procedures used. Comparisons between intracranial and scalp recordings[35,36,37], and findings from magnetoencephalography (MEG) studies[38,39,40,41,42,43], suggest that these ERP components may originate in modality-specific somatosensory areas (i.e., primary and secondary somatosensory areas, SI and SII). In particular, it is has been suggested that the N80 component originates in SI contralateral to the tactile stimulation[35,36,38,40,42,44,45] and the P100 component originates bilaterally from SII[43,46,47]. The source of the N140 component is less understood and may be located in multiple areas[22], including SII and bilateral frontal areas[35,38,39,41,42]. Taken together, the evidence reviewed so far supports that tactile-spatial selection during sustained attention may be mediated by activity in SI and SII. However, it should be noted that while the majority of these ERP studies reported attentional modulations of components generated in SII, the involvement of SI in such effects is less clear. The primary somatosensory cortex receives input from the thalamus, and is thought to encode the location and intensity of tactile stimuli applied to the contralateral hemibody. Somatosensory input is then relayed to SII (although indication of direct projections from the thalamus to SII also exists[48,49]), which is concerned with higher-level integrative processes, such as recognition and memory of tactile information. Thus, the attentional modulation of early ERP components originating in SI may reflect a sensory gain or amplification of the tactile signal prior to full stimulus identification and recognition, while the modulation of activity in SII may indicate that spatial attention influences the subsequent stage of perceptual decision.Furthermore, the involvement of SII in spatial selection between body parts is compatible with the evidence of somatotopic organization in both contralateral and ipsilateral SII[50].

In line with the electrophysiological evidence discussed above, studies using hemodynamic imaging techniques also support the view that sustained spatial attention to a body part can affect activity in somatosensory areas. For example, one study using PET([51]; Experiment 2) reported that maintaining attention to one hand vs. the other during bilateral tactile stimulation resulted in increased rCBF (regional cerebral blood flow) within the postcentralgyrus (corresponding to SI) both when participants had their eyes open and closed during the tactile attentional task[1]. In line with these findings, an fMRI study[52]showed that sustained attention to tactile stimuli (numbers written on a palm) applied to one hand during bilateral stimulation increased activity in the contralateral primary somatosensory area (SI), as well as contralateral sensorimotor regions. Furthermore, similar attentional modulations in contralateral SI were found in a PET study in anticipation of tactile stimuli, but in absence of stimulation[53]. Thus, converging evidence from electrophysiological and hemodynamic studies suggests that sustained tactile-spatial attention modulates tactile processing from early stages. This evidence is supported by a recent study that investigated the effects of sustained spatial attention during bilateral tactile (Braille) stimulation using simultaneous EEG and fMRI recording[2][54]. This study found that early- and mid-latency somatosensory ERP components (P50, N80, and P100), as well as longer latency components (from 175 msec after stimulus onset), were enhanced by tactile-spatial attention. In addition, attention-related fMRI activations were found in SI, SII, the inferior parietal lobe, and some frontal areas. Importantly, attentional modulation of the fMRI signal in contralateral SI was found to be positively correlated with the attentional effects on the P50 component as well as on longer-latency ERP components (from 190 msec after stimulus onset). The findings from this study add to previous evidence that sustained tactile-spatial attention may affect activity in somatosensory areas during initial sensory processing as well as at longer latencies, possibly mediated by re-entrant signals from higher cortical areas. In the next three sections, we examine studies showing that attentional effects in touch may be modulated by multimodal inputs and we discuss the neural mechanisms of these effects.

## CROSS-MODAL LINKS IN SUSTAINED SPATIAL ATTENTION BETWEEN TOUCH AND VISION

In the last 2 decades, there has been a great deal of interest in whether and how mechanisms of spatial attention operate across sensory modalities[55](but see also [56,57] for a discussion of cross-modal and modality-specific attentional effects in the temporal domain). To address this question, researchers have tested whether attending to a spatial location within one sensory modality (e.g., touch) affects the processing of stimuli in a different, task-irrelevant modality presented at that location. Two hypotheses have been put forward. If attentional mechanisms are entirely modalityspecific and segregated, then attentional facilitation should only be found for stimuli in the primary, attended modality. On the other hand, if selective attention is mediated by supramodal mechanisms (i.e., common across modalities), or else if attentional mechanisms in different modalities are "separablebutlinked"[23,58], then attending to a spatial location in a certain modality should result in facilitation of processing also for stimuli in a task-irrelevant modality presented at the attended location, at least to some extent.

A number of studies using different paradigms and methodologies have investigated cross-modal links in sustained spatial attention between touch and vision. For example, in Eimer and Driver's study[18], participants maintained attention to one side of space for an entire experimental block in order to respond to target stimuli (stimuli with a "gap" in the continuous stimulation) among nontargets in the primary modality (e.g., touch) at the attended hand, while ignoring stimuli presented at the unattended hand, as well as all infrequent stimuli in the task-irrelevant modality (e.g., vision). These authors found that attending to a certain spatial location within the tactile modality enhanced not only somatosensory ERPs in response to tactile stimuli presented at that location, but also early visual ERPs elicited by task-irrelevant visual stimuli presented at tactually attended, compared to unattended, locations. However, the attentional modulation of visual ERP components (namely, P1 and N1) was less pronounced when touch

[1] Thedifferences in brain responses between these two conditions (i.e., eyes open vs. closed) are discussed in the section "Effects of Vision of the Body on Sustained Tactile-Spatial Attention".

[2]It should be noted that, in this study, tactile attention was maintained on one hand for short intervals (32 sec), unlike the other studies described in this review where attention was sustained for a longer duration (typically a few minutes).

was the primary modality and visual stimuli had to be ignored compared to when vision was taskrelevant. Thus, sustained attention directed to a location within one sensory modality can spread, to some extent, to another sensory modality in a spatially selective fashion. Interestingly, when vision was the primary modality and tactile stimuli could be entirely ignored, no attentional modulation of somatosensory ERPs was observed in response to infrequent tactile stimuli. This may have a functional advantage in that it would allow us to filter out tactile (proximal) information that may not be relevant when we orient attention to visual or auditory (distal) information (see also [59] for cross-modal attentional links between vision and audition). However, in a follow-up experiment where vision was again the primary modality, but infrequent tactile stimuli now required a response regardless of their spatial location, Eimer and Driver[18] found that somatosensory ERPs were modulated by the location where visual attention was maintained, starting from 140 msecpoststimulus onset. Thus, these results suggest that, different from the effects of tactile attention on vision, touch may be decoupled from visual-spatial attention unless it becomes potentially relevant.

Taken together, these findings support the view that spatial attention operates across sensory modalities. These results further suggest that mechanisms underlying cross-modal spatial attention do not entirely rely on a single supramodal system, but also operate at a modality-specific level, as indicated by larger spatial effects observed for stimuli in the primary (attended) compared to the task-irrelevant modality. A recent computational network model[60] also supports that cross-modal links in spatial attention between vision and touch are mediated by both supramodal and modality-specific mechanisms. In this model, endogenous attention to a stimulus location within one sensory modality (e.g., touch) is implemented as top-down bias input applied to both bimodal and unimodal (e.g., tactile) neurons of the network, within the same hemisphere. Under these conditions, the model predicts a facilitation (reflected in a reduction of the network settling time) for stimuli at the attended compared to the unattended side. These facilitatory effects are stronger in the biased modality (because of the further bias input applied to the unimodal, e.g., tactile, neurons) and somewhat attenuated in the unbiased modality. Crucially, if top-down bias inputs were applied to either the modality-specific or supramodal components only, the model would not be able to predict the spread of attention from one to another modality and the attenuated attentional effects in the secondary modality (respectively) observed in behavioral[61] and ERPs studies[18,62]. Thus, computational evidence supports that spatial attention across modalities operates at multiple levels, resulting in enhanced processing for sensory information presented at the relevant spatial location, particularly, but not exclusively, for the relevant sensory modality.

Another study using PET ([51]; Experiment 1) also supports the view that both modality-specific and supramodal mechanisms are involved in spatial selection of information in one sensory modality. These authors showed that sustained spatial attention to one side of space during bilateral stimulation in one sensory modality (i.e., either touch or vision, in separate blocks) modulated both modality-specific (i.e.,postcentralgyrus and occipital lobe for touch and vision, respectively) and multimodal regions (e.g.,intraparietal sulcus) contralateral to the side of stimulation, with the effects in multimodal areas being present irrespective of the modality of stimulation. These findings suggest that endogenous spatial attention within one modality involves spatial mechanisms that are specific for each sensory modality, as well as mechanisms that are common across modalities. The latter mechanisms may also be responsible for the cross-modal links in spatial attention reported in other studies[18].

Furthermore, fMRI and PET evidence shows that modality-specific visual regions, such as the superior occipital gyrus, are also modulated during sustained spatial attention to touch, although to a lesser extent than during visual attention. Such activations in visual areas during tactile tasks might reflect the notion that spatial reference frames are dominated by vision also in other sensory modalities[63,64,65], possibly because of the high spatial accuracy provided by the visual modality (although it may be noted that in one study by Macaluso et al.[66], the presence of concurrent visual stimuli during tactile selection may have also contributed to activations within visual regions).

Taken together, the findings described in this section show that multimodal mechanisms of spatial selection may be involved also during stimulation in one sensory modality[18,51]. In the next section, we review studies that have attempted to clarify the contribution of vision to tactile-spatial attention; in particular, the specific role of continuous visual input, such as ambient visual-spatial information and vision of the body.

## EFFECTS OF VISION OF THE BODY ON SUSTAINED TACTILE-SPATIAL ATTENTION

The evidence presented in the previous section suggests that mechanisms underlying sustained tactile- and visual-spatial attention operate in synergy. A related, but less explored, issue is whether ambient visual-spatial information and vision of the body can affect tactile-spatial attention mechanisms. In the majority of the studies that have examined the effects of covert tactile-spatial attention, participants had their eyes open throughout the task and their body parts where tactile stimuli were applied (e.g., the hands) were visible to them.

It is known that vision has a profound impact on localization of tactile stimuli in external spatial coordinates[63,64,65,67,68]. However, few studies to date have investigated the role of vision in tactile-spatial selection between body parts. Macaluso and colleagues ([51]; Experiment 2) addressed this issue by comparing cerebral blood flow changes (as indexed by PET) during a sustained tactile-spatial attention task when participants had their eyes open and closed, in separate blocks. As outlined earlier, effects of tactile-spatial attention were found within the postcentralgyrus (i.e., primary somatosensory area, SI) irrespective of whether participants had their eyes open or closed during the task. By contrast, such attentional modulations were observed in the intraparietal sulcus, a multimodal region involved in spatial representation and attention, only when participants had their eyes open. These findings clearly suggest that attention and vision interact in modulating tactile processing. However, from this study, it cannot be resolved whether attentional effects in touch are specifically modulated by ambient visual-spatial information or vision of the body (or both). Indeed, vision of the environment provides a frame of reference to localize events in external coordinates also in modalities other than vision[63,69]. Therefore, it could be expected to help attentional selection compared to when only proprioceptive information is available (i.e., when participants have their eyes closed or are blindfolded). On the other hand, there is evidence that vision of the body, rather than vision of the environment alone, is crucial for proprioceptive localization[70,71,72], which may suggest that the sight of the body could also be important in tactile-spatial selection.

In a series of experiments[19], we showed that vision of the body aids tactile-spatial selection between hands when these are sufficiently far apart, while it impairs within-hand selection. Specifically, in one study, vision of the body was found to facilitate tactile-spatial selection over ambient-visual spatial information[19]. In this study, participants performed a tactile discrimination task, while sustaining their attention to either their right or left hand, under three conditions: full vision, with their hands covered from view, and blindfolded. When both ambient visuospatial information and vision of the hands were available (“full vision”), the somatosensory P100 and N140 components were enhanced for tactile stimuli delivered at attended compared to unattended locations, and these effects were followed by a sustained negativity (Nd) elicited from 200 msec after stimulus onset by tactile stimuli at attended locations. By contrast, under both “covered hands” and “blindfolded” conditions, attentional effects were not present until 200 msec following stimulus onset, when a sustained negativity was observed for attended compared to unattended tactile stimuli (see Fig. 2). Consistent with this pattern of ERP results, shorter reaction times (RTs) were found under full vision compared to when participants’ hands were covered and when participants were blindfolded. Thus, tactile-spatial selection appears to be specifically aided by viewing one’s own body, while visuospatial information *per se* does not seem to facilitate tactile-spatial selection[3].

[3]Moreover, unpublished data from our recent work suggest that visual facilitation of tactile-spatial selection is not affected by different angles of view of the participants’ hands. In particular, attentional ERP modulations were present in the range of the P100 component as well as at later latencies (Nd) irrespective of whether participants’ gaze was directed (a) in peripersonal space, (b) in extrapersonal space, or (c) in a mirror placed in front of them (while the direct view of their hands was prevented).

This is also in line with previous evidence that viewing the stimulated body part, but not aneutralobject placed at the same location (i.e., visuospatial information only), improves tactile-spatialresolution[73].The

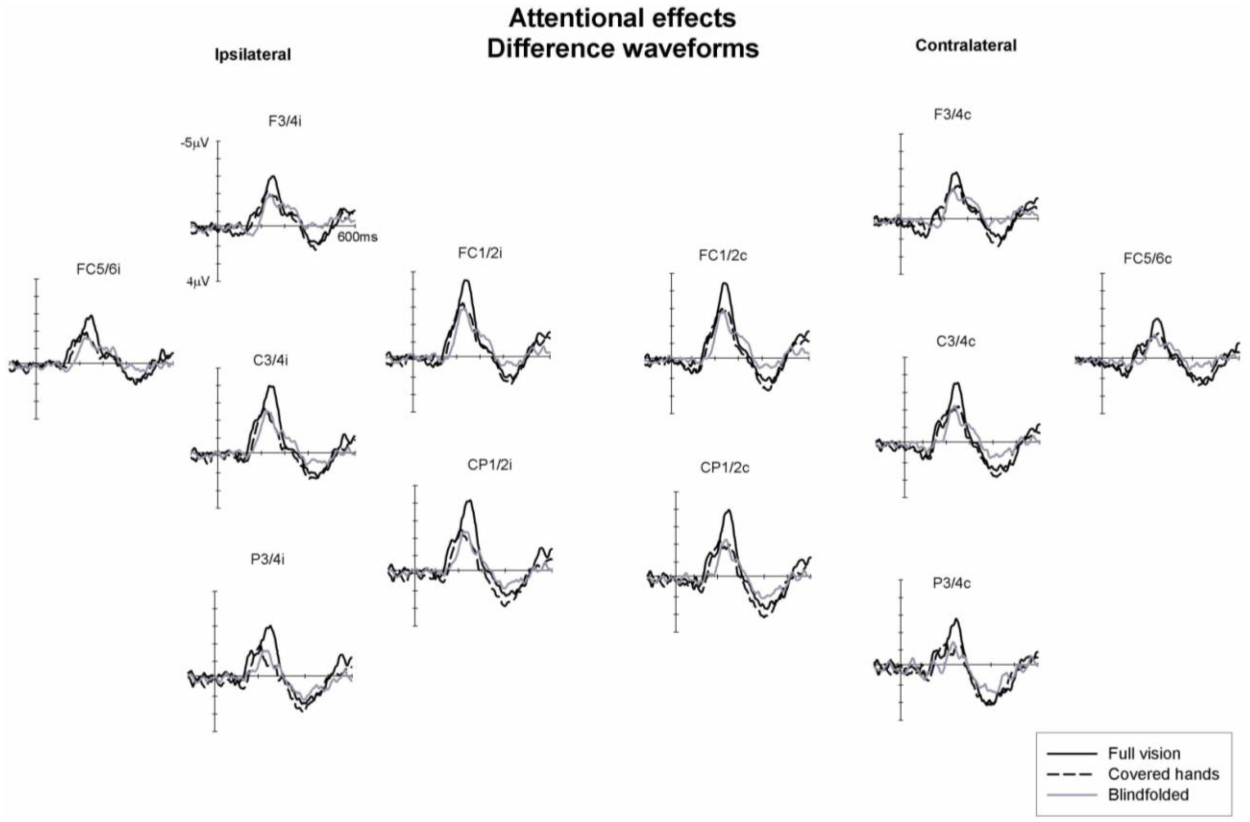

**FIGURE 2.**Visual modulation ofeffects of sustained spatial attention on tactile processing.The figure shows difference ERP waveforms obtained by subtracting ERPs in response to tactile (mechanical) stimuli at attended and unattended locations during the 600-msec interval following stimulus onset, in the conditions "full vision" (*black solid lines*), "covered hands" (*black dashed lines*), and "blindfolded" (*grey solid lines*). The distance from the x axis represents the amplitude of the attentional effects (i.e.,"attended" minus "unattended" ERPs) in each condition. Difference waveforms are shown for electrodes contralateral (right side of the panel) and ipsilateral (left side) to the site of tactile stimulation. In the "full vision" condition,attentional effects were present earlier than in the "covered hands" and "blindfolded" conditions and were more enhanced. See text for further details. (Data from Sambo et al.[19]).

visual modulation of tactile-spatial attention found in our study[19]may result from independent, but converging, projections from frontoparietal brain areas deputed to attentional control and cross-modal visuotactile integration, to somatosensory cortex, or, alternatively, from direct connections between frontoparietal regions involved in attentional and multisensory processing, which would then project back to somatosensory areas.

Furthermore, this facilitation by vision of the body seems to require the hands to be at a distance from each other. Indeed, we found that when participants' hands were placed close together, tactile-spatial selection was not affected by the sight of the hands[74], thus suggesting that vision and hand position may interplay in modulating tactile-spatial selection mechanisms.

On the other hand, we also showed that vision of one's own hand may have a detrimental effect onspatial selection between two adjacent fingers of the same hand (within-hand selection)[74]. In particular, when the participant's hand was covered, sustained spatial attention resulted in an enhancement of the somatosensory P45 and N80 components, while when vision was available,attentional effects were only present at later ERP latencies (i.e., from 200 msec after tactile stimulus onset). Although further investigation is needed to clarify the mechanisms underlying this effect, it is possible that simultaneous vision of adjacent fingers (represented in neighboring regions of SI) may interfere with the filtering of tactile inputs at unattended locations, possibly by modulating lateral interactions in early somatosensory areas (i.e., SI)[75,76]. In line with our findings, it has been shown that in congenitally and early blind people (i.e., with no or limited early visual exposure), effects of within-finger attentional selection were present at earlier stages of somatosensory processing (reflected in an enhancement of the P100 component) compared to sighted participants, where attentional modulations were only observed from 200 msec after tactile stimulus onset[77].

Taken together, the dramatically different effects of vision on between- and within-hand tactile selection mechanisms[19,74] suggest that viewing one's own hands can facilitate attentional selection of tactile stimuli applied to both hands, and thus coded into an external, visuallydominated frame of reference (provided that the hands are sufficiently far apart)[63,78][4]; whereas it impairs (i.e., delays) tactile-spatial selection of individual (adjacent) fingers, which may take place within a somatotopic reference frame[78,80]. In the next section, we review evidence on the contribution of external and somatotopic reference frames on sustained tactile-spatial attention, specifically by examining the influence of body posture and highlighting the role of visual input in such effects.

## EFFECTS OF BODY POSTURE ON SUSTAINED TACTILE-SPATIAL ATTENTION: ANATOMICAL OR EXTERNAL (VISUALLYDEFINED) REFERENCE FRAMES?

In most of the studies reviewed so far, participants attended to their hands while these were held apart in an anatomical (uncrossed) posture. However, in everyday life, our hands assume various positions with respect to each other and to other body parts when we direct attention to them. To date, the effects of hand and, more generally, bodyposture on the mechanisms underlying sustained spatial attention in touch have been little investigated. In this section, we will discuss evidence that changes in body posture affect mechanisms underlying (1) sustained spatial attention in touch and (2) cross-modal links in spatial attention between touch and vision.

First, it has been shown that crossing the hands over the body midline abolished or reversed the attentional modulation of somatosensory ERPs observed under anatomical posture[20,62]. That is, when participants' hands were uncrossed, sustaining (tactile) attention to one hand resulted in enhanced somatosensory ERP components (i.e., N80 and N140, as well as the later Nd); by contrast, when participants assumed a crossed posture, such attentional modulations were absent or tended to be reversed (i.e., larger amplitudes were observed for tactile stimuli delivered at the unattended hand). The latter result suggests that more attention was allocated to the currently "unattended" hand when this was held in the hemispace, where typically the other, to-be-attended hand is seen. Altogether, these findings also indicate that crossing the hands over the midline disrupts tactile-spatial selection processes, similar to what has been shown for other tasks (e.g., temporal order judgment[65,81]). With crossed hands, the anatomical and external, visuallydefined spatial reference frames for coding body locations are in conflict (e.g.,[63,82]; see also discussion in the section "Effects of Vision of the Body on Sustained Tactile-Spatial Attention), which as a result may interfere with spatial attentional selectivity in touch. Crucially, this also suggests that when the hands are held in an anatomical posture,

[4]See also Gillmeister and Forster[79] for evidence of facilitation by vision of the body in a nonspatialtactile-attention task.

both somatotopic (anatomical) and external reference frames are likely to be used to direct attention to body locations.

The use of anatomical and external reference frames in sustained tactile-spatial attention has also been shown when directing attention to anatomically distant body parts (e.g., hands and feet)[20]. For example, Heed and Roder[20] used a spatial gradient paradigm whereby participants adopted an uncrossed- and a crossed-hands posture, in separate blocks, while their feet were placed near their hands. These authors showed that attending to a certain body part (i.e., one's own foot) also affected tactile processing at a different body site (i.e., the hands) as a function of the distance from the attended body part. In particular, attentional modulations of somatosensory ERPs were observed at early, perceptual stages of processing (100–140 msecpoststimulus onset) both when the stimulated hand was placed nearas opposed to far from the attended foot, irrespective of whether this spatial proximity resulted from an uncrossed or a crossed posture, and when the stimulated hand was ipsilateral as opposed to contralateral to the attended foot, irrespective of spatial proximity. These findings suggest that attention to a location on the body may spread to other body parts that are not contiguous (e.g., from the feet to the hands). Importantly, such attentional effects may reflect two distinct mechanisms, one involving a spatial remapping of body locations in external ("visual") coordinates and the other relying on anatomicallydefined coordinates. This indicates that both anatomical information and information on one's body posture play a role when directing attention to a certain body location. However, because an interaction between external spatial proximity and anatomical congruence was not found in Heed and Roder's study[20], this suggests that these two mechanisms for tactile-spatial attention may operate in parallel.

Second, the role of body posture in sustained tactile-spatial attention has also been investigated using a cross-modal paradigm. For example, Eimer and colleagues[62] used a method similar to that used in Eimer and Drivers[18] (see section "Crossmodal Links in Sustained Spatial Attention between Touch and Vision") whereby participants performed a tactile discrimination task, while in some of the trials task-irrelevant visual stimuli were presented near the hands instead of the tactile stimuli, at tactually attended and unattended locations, under both uncrossed and crossed postures. While the results for uncrossed-hands trials confirmed those reported by Eimer and Driver[18], the crucial new finding was that, under crossed posture, attentional modulations were observed on the visual N1 and P2 components for visual stimuli presented in the same hemispace where the tactually-attended hand was held, but not for visual stimuli ipsilateral to the tactile stimuli in terms of hemispheric projections. This indicates that such cross-modal attentional links are mediated by *external* spatial coordinates after changes in hand posture are taken into account. Indeed, these findings rule out the possibility that cross-modal links in spatial attention result from spread of activation between modality-specific areas within the same hemisphere (contralateral to the attended side; i.e., hemispheric-activation hypothesis[83,84]), as this model does not account for the effects following postural changes.

To summarize, in this section, we have examined evidence, first, that both anatomical and external frames of reference may be used to direct attention to body locations, and, second, that cross-modal attentional links between touch and vision are mediated by an external-spatial frame of reference where hand posture is taken into account. As outlined in previous sections, an external-coordinates system for tactile-spatial processing has been shown to rely on early visual experiences[63,82,85]. Moreover, it has been suggested that such a reference system is used by sighted individuals, but not by congenitally blind, to direct attention to body parts on a trial-by-trial basis (i.e., transient attention)[85]. Although the neural underpinnings of these postural effects on spatial attention have not been yet determined, these may involve an interplay between visual, tactile, and proprioceptive inputs, possibly mediated by top-down projections from frontoparietal regions to somatosensory areas[86]. In line with this account, bimodal (visual and tactile) neurons have been documented in these higher-order areas (e.g., the premotor cortex and the intraparietal sulcus[21,87,88]; see also [89,90] for fMRI evidence in humans), a percentage of which have their visual RFs anchored to a body part (i.e., the hands) and move when the body part moves in space, so that they maintain spatial alignment with the tactile RFs[88]. Furthermore, frontal and parietal regions are known to play a role in attentionalselection[5,91,92]. The latencies of the effects discussed in this section (i.e., overlapping with the somatosensory components N80, N140, and Nd for the effects of posture on tactile-spatial attention; and the visual N1 and P2 components for the cross-modal spatial-attentional links between vision and touch) are compatible with the involvement of both modality-specific and multimodal mechanisms. Future studies may address the contribution of ambient visual-spatial information and vision of the body to postural modulations of tactile-spatial selection during sustained attention.

## CONCLUSIONS

Increasing evidence shows that sustained spatial attention to touch can affect activity in early, modality-specific areas (i.e., SI and SII). Together, these findings support the notion that attentional selection operates at the level of perceptual processing, i.e., as an “early filter”, possibly enhancing tactile processing at relevant spatial locations and filtering out tactile information at irrelevant locations[51,75,93]. This evidence is also in line with effects of spatial-selective attention reported in other sensory modalities, such as vision and audition. However, while the modulation of the respective primary sensory cortices by visual and auditory spatial attention has been confirmed several times[94,95,96,97], the involvement of SI in tactile-spatial selection is still unclear and has been only reported in some studies.

Although the specific mechanisms underlying tactile-spatial attention are not fully understood, evidence suggests that spatial-selective attentional effects may result from descending signals from frontoparietal regions involved in spatial attentional selection across modalities to somatosensory areas[5,86]. Thus, spatial selection in touch may recruit both multimodal and modality-specific areas, similar to other sensory modalities[59,98,99]. This cortical network would allow flexible and efficient spatial selection within and across modalities, possibly underpinning cross-modal links in spatial attention[23].

Furthermore, recent studies have shown that vision of the body and body posture can affect neural mechanisms underlying sustained tactile-spatial attention. First, viewing the body has been previously shown to enhance tactile processing independently of spatial orienting[73,74,100,101,102,103]. Thus, the finding that tactile-spatial selection is also improved by vision of the body may suggest that vision and attention interact to increase saliency of tactile events. Moreover, the evidence that body posture is taken into account when directing attention to a location on the body is in agreement with the notion that tactile stimuli are encoded not only in somatotopic coordinates, but also in an external, visuallydefined reference frame[63,67,82,85].
